# Absorption and Fluorescence Emission Investigations on Supramolecular Assemblies of Tetrakis-(4-sulfonatophenyl)porphyrin and Graphene Quantum Dots

**DOI:** 10.3390/molecules29092015

**Published:** 2024-04-27

**Authors:** Mariachiara Sarà, Salvatore Vincenzo Giofrè, Salvatore Abate, Mariachiara Trapani, Rosaria Verduci, Giovanna D’Angelo, Maria Angela Castriciano, Andrea Romeo, Giovanni Neri, Luigi Monsù Scolaro

**Affiliations:** 1 Dipartimento di Scienze Chimiche, Biologiche, Farmaceutiche ed Ambientali, University of Messina, V.le F. Stagno D’Alcontres, 31, 98166 Messina, Italy; mariachiara.sara@studenti.unime.it (M.S.); salvatorevincenzo.giofre@unime.it (S.V.G.); salvatore.abate@unime.it (S.A.); mcastriciano@unime.it (M.A.C.); anromeo@unime.it (A.R.); 2 CNR—ISMN Istituto per lo Studio dei Materiali Nanostrutturati c/o Dipartimento di Scienze Chimiche, Biologiche, Farmaceutiche ed Ambientali, University of Messina, V.le F. Stagno D’Alcontres, 31, 98166 Messina, Italy; mariachiara.trapani@cnr.it; 3 Dipartimento di Scienze Matematiche e Informatiche, Scienze Fisiche e Scienze della Terra, University of Messina, V.le F. Stagno D’Alcontres, 31, 98166 Messina, Italy; rosaria.verduci@unime.it (R.V.); giovanna.dangelo@unime.it (G.D.); 4 Dipartimento di Ingegneria, University of Messina, Contrada di Dio, 98158 Messina, Italy; giovanni.neri@unime.it

**Keywords:** porphyrins, graphene quantum dots, supramolecular adducts, fluorescence emission

## Abstract

The one-pot synthesis of N-doped graphene quantum dots (GQDs), capped with a positively charged polyamine (trien), has been realized through a microwave-assisted pyrolysis on solid L-glutamic acid and trien in equimolar amounts. The resulting positively charged nanoparticles are strongly emissive in aqueous solutions and are stable for months. The interaction with the anionic tetrakis(4-sulphonatophenyl)porphyrin (TPPS_4_) has been investigated at neutral and mild acidic pH using a combination of UV/vis absorption spectroscopy together with static and time-resolved fluorescence emission. At pH = 7, the experimental evidence points to the formation of a supramolecular adduct mainly stabilized by electrostatic interactions. The fluorescence emission of the porphyrin is substantially quenched while GQDs remain still emissive. On decreasing the pH, protonation of TPPS_4_ leads to formation of porphyrin J-aggregates through the intermediacy of the charged quantum dots.

## 1. Introduction

Graphene is a single layer of graphite and consequently is an atomically flat monolayer with a hexagonal or honeycomb arrangement of carbon atoms. This 2D material was discovered in 2004 [1] and its importance has grown prominently with the investigations on its peculiar chemical and physical properties that fostered a wide variety of applications [2,3]. In the world of graphene based nanomaterials, graphene quantum dots (GQDs) are interesting for their optical and electronic properties [4], which have been exploited in a series of applications spanning different fields such as bio-imaging [5,6,7,8,9], sensing [10,11,12,13], and energy conversion [14,15,16]. The presence of a variety of oxygen-containing functional groups (e.g., COOH, OH, CHO, or OCH_3_) contributes to enlarge the chemical reactivity of these nanomaterials and especially to largely modulate their electronic and optical properties [17]. Fluorescence is a peculiar feature emerging in these nano-systems and many experimental parameters, including the choice of the carbon precursor and the synthetic route, the size, and the presence of doping atoms (e.g., N, S, P, or B), contribute to modulate consistently the energy of the emission bands [18,19,20]. Among the plethora of reported methodologies of synthesis, both the top-down and the bottom-up approaches have been reported. The first approach uses graphite as precursor material and exfoliation, laser ablation, or electrochemical methods to obtain the nanomaterial. The second strategy starts from rather simple organic compounds through pyrolysis, hydrothermal or solvothermal treatments, or microwave-assisted decomposition [15,21,22]. These techniques mostly afford GQDs that are soluble in water, and that can be stabilized and modified conveniently through a variety of capping reagents [23]. The access to rather complex supramolecular systems, in which GQDs are assembled with other molecular components, can be achieved by non-covalent interactions, e.g., electrostatic, hydrogen bonding or hydrophobic interactions. In this respect, porphyrins are an important class of building blocks that have found large-scale application to obtain nano-architectures where the manifold properties of such compounds can be used for a variety of applications [24,25,26,27]. The formation of supramolecular adducts between GQDs and porphyrins [28,29,30,31,32,33,34] and the occurrence of photo-induced electron transfer from the macrocyclic chromophores to the carbon nanoparticles has been reported in organic [35] and aqueous solvent [36]. These kind of supramolecular adducts have been also exploited to build very sensitive metal ion sensors [37,38,39]. Indeed, the ability of porphyrins to bind a large number of metal ions is enhanced when these species are bound to graphene materials [40].

On these bases, it would be interesting to investigate the interaction between positively charged GQDs and the water soluble negatively charged tetrakis(4-sulphonatophenyl)porphyrin (TPPS_4_). This porphyrin is a tetra-anionic species under neutral pH and has already demonstrated its ability to self-assemble with many different complex systems, including polymers [41,42,43], polypeptides [44,45], proteins [46,47,48], inorganic nanoparticles [49,50], carbon nanotubes [51], and carbon dots [52]. To the best of our knowledge, only two reports treat the interaction between GQDs and this specific porphyrin: (i) the first deals with the self-assembling process of the neutral TPPS_4_ on different GQDs bearing positively charged diamines at neutral pH [36], and (ii) the second describes the formation of J-aggregates on similar GQDs with a specific mixing protocol [52]. Here we report a new synthesis of GQDs using microwave-assisted pyrolysis of glutamic acid and triethylenetetramine tetrahydrochloride (*trien*) as precursors. The obtained nanoparticles indicate a positively charged surface, due to the presence of residual ionizable cationic amino-groups, and some residual *trien* as a capping reagent, and consequently can interact quite efficiently with the negative TPPS_4_. The resulting adduct leads to a deactivation of the fluorescence emission from the porphyrin fluorophore, probably through a photo-induced electron transfer. On decreasing the pH, the nature of the interaction substantially changes, being the diacid form of the porphyrin responsible, with the intermediacy of the GQDs, for the formation of porphyrin J-aggregates. We anticipate that *trien* plays a role to foster the electrostatic binding of negatively charged porphyrins to the GQDs and to trigger the easy formation of J-aggregates at mild acidic pH conditions and characterized by quite broad electronic spectra. This point is important as it marks the difference with previous reports and opens the way to the design of nanoassembled systems with peculiar spectroscopic features.

## 2. Results

### 2.1. Synthesis and Spectroscopic Characterization of Graphene Quantum Dots (GQDs)

**Scheme 1:**
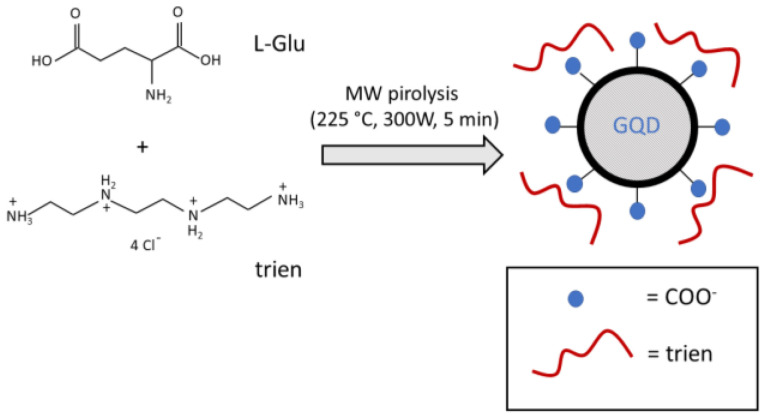
Sketch diagram of the MW-assisted synthesis of GQDs used in this study.

Graphene quantum dots (GQDs) have been synthesized by microwave-assisted pyrolysis (225 °C, 300 W, 5 min) of solid L-glutamic acid as organic precursor in the presence of triethylenetetramine tetrahydrochloride (*trien*) in equimolar amount (Scheme 1). The dark brown powder has been treated with de-ionized water, obtaining a brown solution that was purified initially by filtration and eventually by dialysis. 

**Figure 1:**
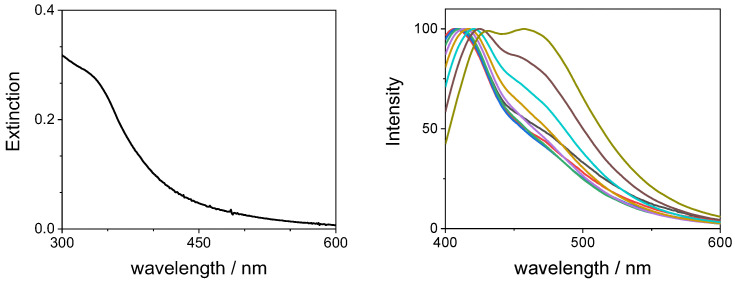
Spectroscopic features of an aqueous solution of GQDs (0.14 mg/mL): UV/Vis absorbance spectrum (**left panel**) and normalized fluorescence spectra (**right panel**, excitation wavelength from 310 to 390 nm).

**Figure 2:**
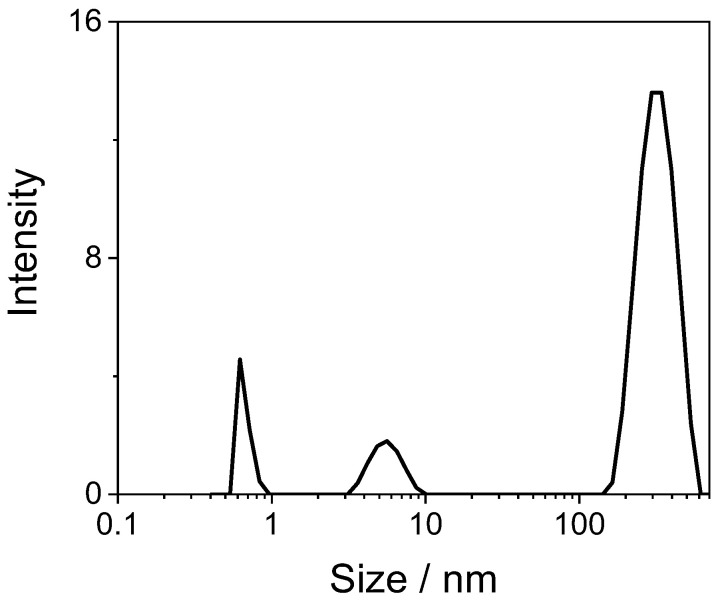
Distribution of particle size measured on an aqueous solution of GQDs (0.14 mg/mL) through the dynamic light scattering technique.

Figure 1 (left panel) shows a typical UV/Vis spectrum of a diluted sample of GQDs in water that exhibits an unstructured absorption profile with a shoulder at 330 nm. These samples are light blue emissive, and the corresponding spectra display two emission bands: (i) one is centered at 458 nm and its position does not depend on the excitation wavelength and (ii) a second one whose intensity and position (407–432 nm) are dependent on the excitation wavelength. Figure 1 (right panel) reports the normalized fluorescence emission spectra corresponding to excitation from 310 up to 390 nm. The observed dependence of the luminescence spectra upon excitation wavelength could be explained by the presence of particles having different sizes in solution [18]. Dynamic light scattering (DLS) measurements reveal the presence of particles with different sizes, from very small (0.8 ± 0.2 nm) and medium-small (5.8 ± 0.9 nm) up to quite large (260 ± 40 nm) (Figure 2). These latter ones are probably due to clustering of the medium-small particles. 

The presence of the capping *trien* is probed by the positive zeta-potential (+24 mV) measured on these samples at pH = 7. Usually, GQDs obtained from neat L-glutamic acid or other similar organic sources are negatively charged due to the presence of residual ionized carboxylate groups on the periphery of the nanoparticles [53]. Under neutral pH conditions, the *trien* polyamine should be partially protonated, electrostatically interacting with the negatively charged COO^−^ groups and responsible for the overall positive charge of the GQDs.

The aqueous solutions of GQDs remain quite stable in terms of size and emission properties for at least three months.

**Figure 3:**
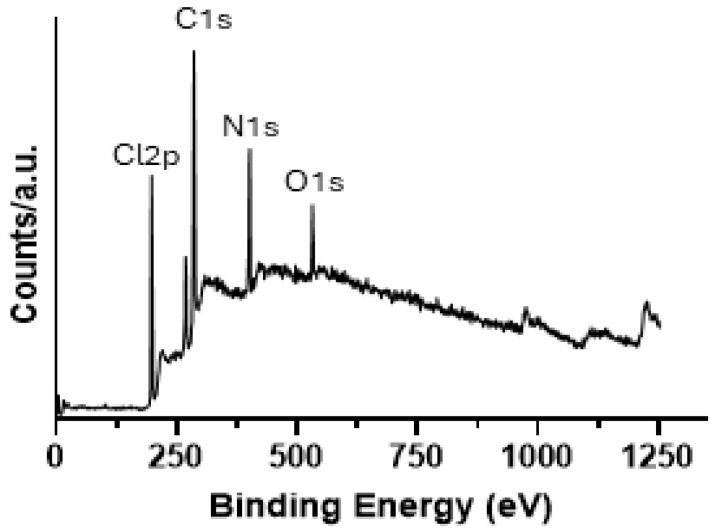
XPS survey spectrum for a blank mixture containing the precursors before the pyrolysis procedure.

Further characterization of the GQDs has been performed through X-ray photoelectron spectroscopy (XPS). The survey spectra (Figure 3) indicate that the samples contain mainly C, N, Cl, and O elements. The surface composition in weight % is reported in Table 1 for samples containing a mixture of precursors before the pyrolysis and the GQDs were investigated.

**Figure 4:**
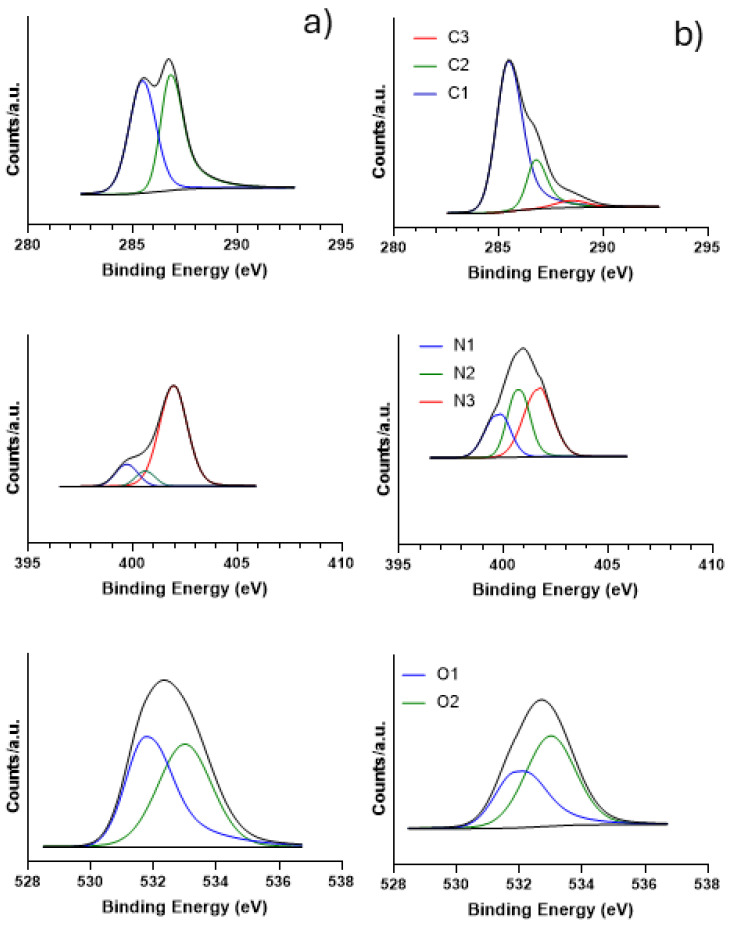
XPS spectra for the C1s, N1s, and O1s regions for (**a**) the sample before pyrolysis and (**b**) for GQDs.

Figure 4a reports the fitted spectra for the blank sample. The C1s region shows: (i) an asymmetric peak at 285.46 eV (C1 component in blue) that is attributed to the C–N/C–O species, exhibiting large FWHM that indicates also the presence of C atoms in sp^3^ bonding or of highly defective sp^2^ carbon phase; (ii) a second peak at 286.77 eV (C2 component in green), which is attributed to C=N/C=O [54] or C–Cl species [55]. The N1s core level region shows a first small component at 399.68 eV assigned to N-H species (N1 component in blue), a second one at 400.66 eV assigned to C–N amide or C=N–H species (N2 component in green) [54], and a third peak at 401.80 eV attributed to quaternary N (N3 component in red) [56]. The O1s region displays a first peak at 531.87 eV (O1 component in blue) attributed to C–N–O and the second peak at 532.99 eV (O2 component in green) assigned to C–OH specie [57].

The XPS spectra of GQDs are reported in Figure 4b. In the C1s core level, the component C2 is less intense, passing from 48.64% in the blank sample to 20.39% in the treated one. Moreover, a small component appears at 288.50 eV (C3 component in red) assigned to conjugated N–C=N bond, including quaternary N species [56]. The most intense peak is the C1 component that increases from 51.36% to 75.67% in the treated sample indicating an increase in the highly defective sp^2^ carbon phase. Concerning the N1s levels, the components at higher binding energy (BE) decrease to the detriment of those at lower BE. Generally, all the components are shifted at higher BE, indicating the interaction of the N species with carbon. Both the components N1 and N2 increase, while the third component N3 decreases from 78.39% to 42.99% with respect to the blank sample, in line with the reduced amount of Cl.

Figure S1 reports the Cl2p region for the blank sample. A main peak is observed at 198.13 eV and assigned to ammonium chloride in line with the IST X-ray Photoelectron Spectroscopy Database. After pyrolysis, the peak retains the same BE even if it decreases from 29.43 (blank sample) to 11.84 wt%, as reported in Table 1.

**Figure 5:**
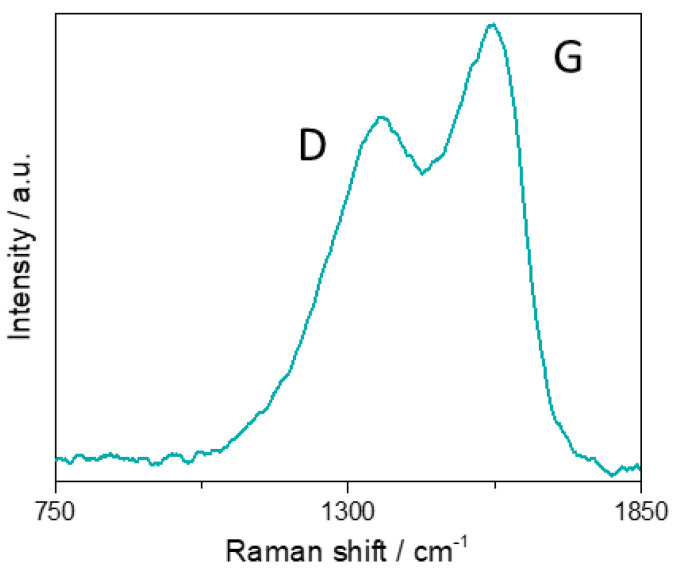
Raman spectrum of a GQDs solid sample.

The Raman spectrum of solid GQDs (Figure 5) shows the two typical bands centered at 1567 cm^−1^ (G band) and 1363 cm^−1^ (D band), in agreement with literature data [53]. The G band arises from the in plane sp^2^ C–C stretching vibrations in the graphene nanostructures. The D band reveals the breakdown of the sp^2^ network caused by the formation C–C- sp^3^-bonds.

**Figure 6:**
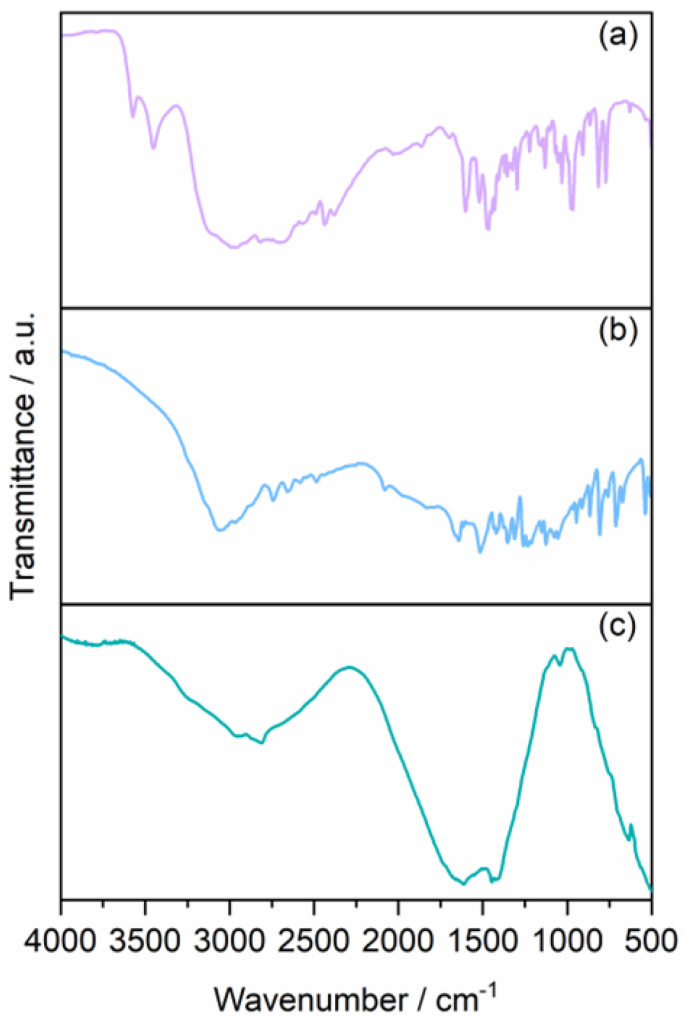
IR spectra of (**a**) trien, (**b**) L-glutamic acid, and (**c**) GQDs as solids onto silicon windows.

FT-IR spectroscopy has also been exploited to characterize the GQDs. The spectra of the two solid precursors have been compared with that of the solid sample after pyrolysis (Figure 6). The assignment of the peaks has been achieved by comparison with the data reported by Wu et al. [53]. The large band at 3000 cm^−1^, together with the strong peak in the region 1700–1500 cm^−1^, is ascribed to the C=C stretching vibrations of graphite. The peak at 1456 cm^−1^ is also clearly visible in the trien spectrum and could be assigned to methylene groups’ bending vibration. A rather broad peak appears at 2600–2870 cm^−1^, probably related to the O-H stretching mode of the carboxylic residues in the GQDs, while the wide peak extending in the 1560–1730 cm^−1^ arises from the combination of the C=O and the amide stretching modes. Finally, the broad peak located around 4000–3600 cm^−1^ and the peak at 3270 cm^−1^ are assigned to the N–H stretching of the amide and amino groups, respectively. 

### 2.2. Interaction of Graphene Quantum Dots (GQDs) with TPPS_4_ Porphyrin

**Scheme 2:**
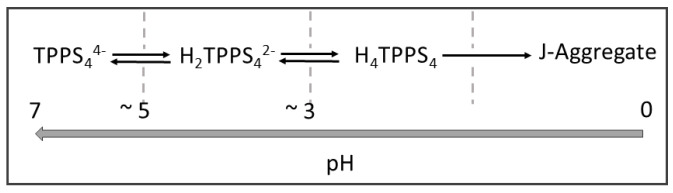
Various forms of TPPS_4_ porphyrin and formation of J-aggregates depending on the pH of the solutions.

As a function of the solution pH, TPPS_4_ porphyrin undergoes different protonation steps, forming species with different global charge [58]. At neutral pH, the porphyrin is a tetra-anion (TPPS_4_^4−^), being the four sulphonate groups fully ionized. The pK_a_ for the addition of protons to the two nitrogen atoms in the central core is 4.9 [59]; therefore, the di-anion (H_2_TPPS_4_^2−^) forms at pH values lower than 3.9. Decreasing the pH below 3, protonation of two sulphonate groups leads to a neutral zwitter-ion (H_4_TPPS_4_) [58]. This species is the precursor to J-aggregates that originate through the supramolecular assembling process of the monomers mediated by non-covalent interactions (Scheme 2) [58]. 

**Figure 7:**
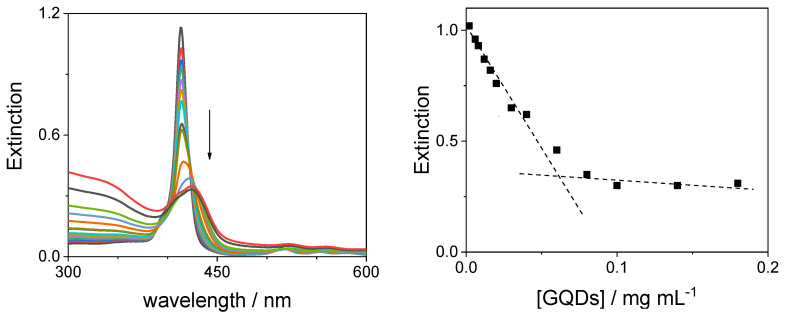
UV/Vis extinction spectral changes (**left panel**) and extinction at 413 nm (**right panel**) during the titration of TPPS_4_^4−^ with GQDs at neutral pH (the arrow marks the increasing GQD concentration). The dashed lines are a visual reference. Experimental conditions: [TPPS_4_^4−^] = 3 μM; [GQDs] = 0–0.18 mg/mL; phosphate buffer 1 mM, pH = 7; T = 298 K; cell path length 1 cm.

Upon addition of increasing amounts of GQDs to an aqueous solution of TPPS_4_^4−^ at pH 7, the UV/Vis spectra show the gradual increase in the absorbance in the UV region around 330 nm, due to the increasing concentration of the carbon nanoparticles. At the same time, the B-band of the porphyrin undergoes a substantial hypochromicity (~70%) and bathochromic shift (Δλ = +11 nm), moving from 413 to 424 nm (Figure 7, left panel). An isosbestic point is clearly identifiable at 420 nm and it is maintained up to a concentration 0.04 mg/mL of GQDs. Above this concentration, the isosbestic point is not retained, probably due to the distortion introduced in the spectra by the increasing scattered light, together with the increasing contribution of absorption by the added GQDs. The changes in the extinction at 413 nm upon titrating TPPS_4_^4−^ with the GQDs are shown in Figure 7 (right panel). The data display a break-point at about 0.06 mg/mL of GQDs. An inspection of the Q-band region also reveals that these spectral features are affected by red shifts. As far as their intensities are concerned, the presence of a light scattering component makes it difficult to evaluate precisely their relatively small changes. The spectral changes are accompanied by a consistent broadening of the B-band. These experimental findings suggest a change in the microenvironment experienced by the porphyrin monomer and indicate the progressive binding to the GQDs together with the occurrence of self-aggregation of the macrocycles. 

The shift of the B-band towards lower energy indicates the formation of J-type coupling among the chromophores [60]. This finding is different with respect to what was reported for a similar system, where a hypsochromic shift has been observed, probably due to formation of H-type dimers [52]. Also, the amount of hypochromicity in the system investigated here is more than double with respect to carbon nanodots derived from ethylenediamine in the organic precursor [36]. 

**Figure 8:**
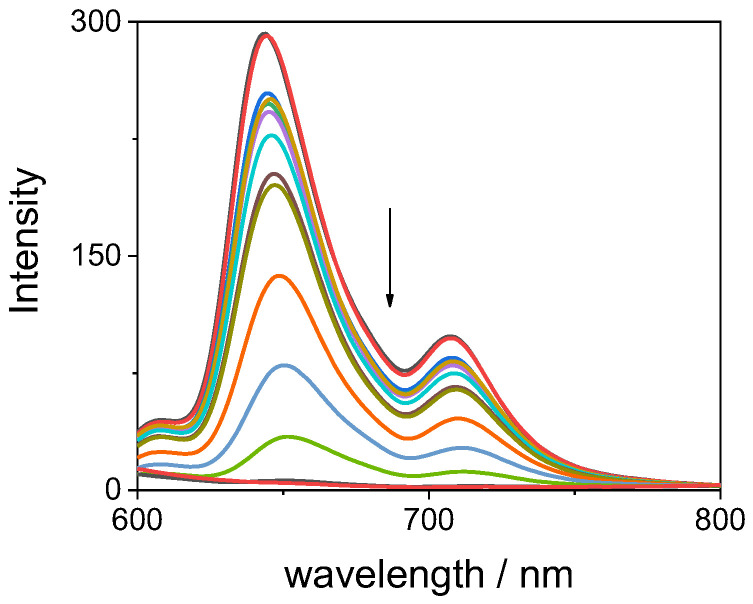
Fluorescence emission spectral changes during the titration of TPPS_4_^4−^ with GQDs at neutral pH (the arrow marks the increasing GQD concentration). Experimental conditions: [TPPS_4_^4−^] = 3 μM; [GQDs] = 0–0.18 mg/mL; phosphate buffer 1 mM, pH = 7; T = 298 K; cell path length 1 cm; excitation wavelength 420 nm (isosbestic point in the UV/Vis titration).

**Figure 9:**
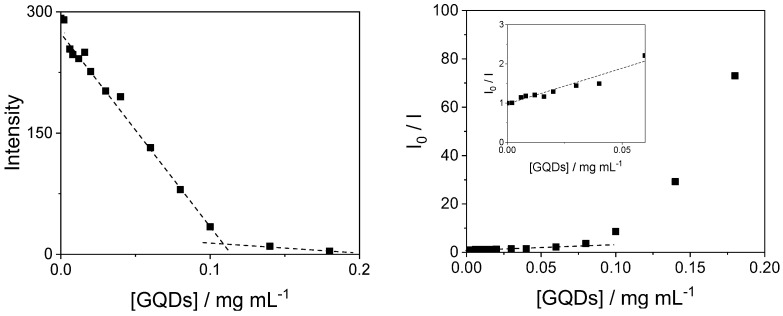
Fluorescence emission intensity changes at 645 nm during the titration of TPPS_4_^4−^ with GQDs at neutral pH (**left panel**). The dashed lines are a visual reference. Stern–Volmer plot of I_0_/I as function of increasing GQD concentration (**right panel**), together with an expansion of the low GQD concentration region (inset of **right panel**). Experimental conditions: [TPPS_4_^4−^] = 3 μM; [GQDs] = 0–0.18 mg/mL; phosphate buffer 1 mM, pH = 7; T = 298 K; cell path length 1 cm; excitation wavelength 420 nm (isosbestic point in the UV/Vis titration).

The TPPS_4_^4−^ porphyrin in aqueous solution is emissive and the relative emission spectra show two well-defined bands at 645 and 707 nm (Figure 8). Upon addition of increasing amounts of GQDs, the emission is progressively quenched, and it is possible to observe a slight red shift in the position of the bands (from 645 to 651 nm and from 707 to 711 nm). Figure 9 (left panel) reports the progressive quenching of the emission fluorescence intensity during the titration of the porphyrin with the GQDs. A break-point is clearly visible upon addition of 0.1 mg/mL of GQDs. In contrast with carbon quantum dots reported in the literature [36], in our system the quenching effect is almost complete. A Stern–Volmer plot of the ratio I_0_/I (where I_0_ and I are the fluorescence emission in the absence and in the presence of GQDs, respectively) shows an almost linear behavior for GQD concentration up to 0.06 mg/mL (inset in Figure 9 right panel), suggesting a dynamic quenching mechanism. From these data a value for the Stern–Volmer constant K_D_ = 13.7 ± 0.2 (mL/mg) can be calculated. Above this concentration, a large deviation is readily observable that could be ascribable to either aggregation of porphyrin or static quenching. Time-resolved fluorescence measurements show that, in agreement with the literature [61], the intensity profile for the free base porphyrin TPPS_4_^4−^ in aqueous solution can be described essentially by a mono-exponential decay with a τ_1_ = 10.0 ± 0.1 ns (A = 97%). A small, fast-decaying contribution is also detectable in our solutions with a τ_2_ = 1.1 ± 0.1 ns (A = 3%), probably due to a tiny quantity of porphyrin oligomers in solution (Table S1). Upon increasing the GQD concentration, the τ_0_/τ_1_ ratio (where τ_0_ and τ_1_ are the fluorescence lifetime emission in the absence and in the presence of GQDs, respectively) remains almost constant to unit, probably confirming a static quenching mechanism [62]. Actually, the relative amplitudes of the two lifetime contributions show a matching and parallel change: upon increasing GQD concentration, τ_1_ values slightly decreases (from 97 to 86%), while τ_2_ increases (from 3 to 14%) (Table S1). Our results are similar to those reported for the interaction of the TPPS_4_ porphyrin with other positively charged carbon dots [36]. Lifetime-resolved fluorescence anisotropy measurements allow deriving information about the dynamics of the fluorophore in solution. The rotational correlation times τ_r_ can be derived from Perrin equation [62], and for TPPS_4_^4−^ porphyrin, the measured value (τ_r_ = 0.72 ns) is in close agreement with a monomeric species in solution [61]. Upon addition of increasing amounts of GQDs, the values of τ_r_ display only a slight increase (~25%), suggesting that the porphyrins are interacting with GQDs, even if they are substantially free to rotate in solution (Table S1). This could imply that electrostatic interactions between positively charged *trien* amino groups of the surface of GQDs keep the negatively charged TPPS_4_^4−^ close to the carbon nanoparticles, although without blocking them completely. Indirect evidence of the role played by the positively charged *trien* capping reagent is given by titrating the positively charged tetrakis(N-methyl-pyridinium-4-yl)porphyrin (TMpyP(4)) with our GQDs. In this case, upon adding increasing amounts of the graphene nanoparticles to a porphyrin solution, the UV/Vis spectra do not indicate any substantial change (Figure S2), while the emission fluorescence is only slightly perturbed (Figure S3). These observations confirm that *trien* is important in driving a strong electrostatic contact with the charged substituent groups on the porphyrins.

**Figure 10:**
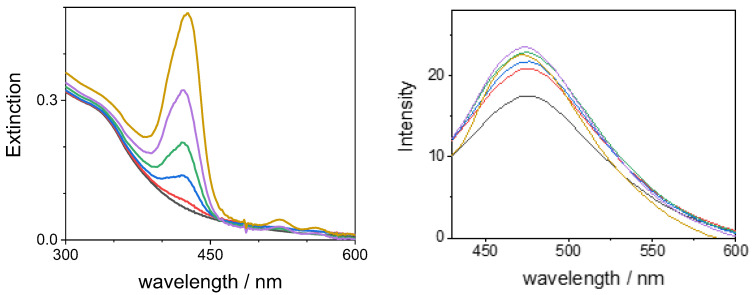
UV/Vis absorbance spectral changes (**left panel**) and fluorescence emission spectra exciting at λ = 410 nm (**right panel**) during the titration of GQDs (black line) with TPPS_4_^4−^ at neutral pH. Experimental conditions: [TPPS_4_^4−^] = 0.07 µM, red line; 0.42 µM, blue line; 0.9 µM, green line; 1.8 µM, purple line; 3 µM, yellow line; [GQDs] = 0.14 mg/mL; phosphate buffer 1 mM, pH = 7; T = 298 K; cell path length 1 cm.

When an aqueous solution of GQDs is titrated with TPPS_4_^4−^ porphyrin at neutral pH, the corresponding UV/vis spectral changes are reported in Figure 10 (left panel). The UV region of the spectra is only slightly modified in intensity, probably due to a moderate increase in absorbance due to the less intense electronic transitions of the porphyrin at these wavelength values. No visible change in the position of the absorption band of the GQDs is detectable. On increasing the porphyrin concentration, a B-band grows at 424 nm, corresponding to the position observed for the direct titration of TPPS_4_^4−^ with GQDs. The fluorescence spectra obtained by exciting the system at 410 nm show a profile with a band at 470 nm (where the porphyrin does not emit) that is relative to the emission of the GQDs (Figure 10, right panel). After correcting the emission spectra for the filter effect, the measured emission profiles are almost unaffected by the increase in the concentration of porphyrin (Figure 1, right panel). A similar behavior is shown when the samples are excited at 330 nm. Time-resolved fluorescence measurements reveal that the intensity profile (λ_exc_ = 390 nm; λ_em_ = 474 nm) for the free GQDs in aqueous solution at neutral pH can be described by three exponential decays with τ_1_ = 1.8 ± 0.1 ns (A_1_ = 25%), τ_2_ = 5.4 ± 0.2 ns (A_2_ = 61%), and a longer time τ_3_ = 15.4 ± 0.1 ns (A_3_ = 14%). On increasing the porphyrin amount in solution, these values remain essentially unaltered (see Table S2). These experimental findings suggest that no effective energy transfer occurs from the GQDs to porphyrins that are bound to the surface of the nanoparticles.

**Figure 11:**
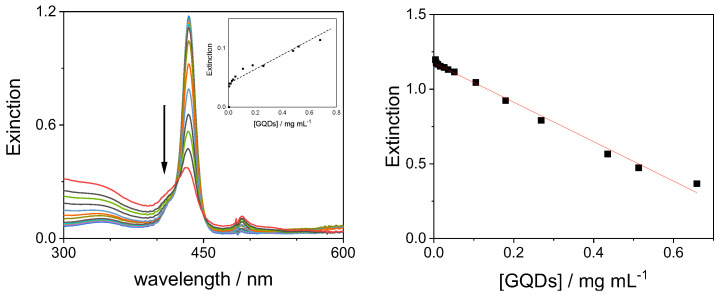
UV/Vis extinction spectral changes (**left panel**) and the decrease in extinction at 434 nm (**right panel**) during the titration of H_2_TPPS_4_^2−^ with GQDs at pH = 3 (the arrow marks the increasing GQD concentration). In the inset (left panel), the increase in the band at 491 relative to the J-aggregate as function of GQD concentration. Experimental conditions: [H_2_TPPS_4_^2−^] = 3 μM; [GQDs] = 0–0.78 mg/mL; [HCl] = 1 mM, pH = 3; T = 298 K; cell path length 1 cm.

By progressively titrating an aqueous solution of TPPS_4_^4−^ porphyrin with GQDs at pH = 3, the corresponding UV/vis spectral changes are rather different with respect to neutral pH and are displayed in Figure 11 (left panel). At this pH, the porphyrin is present as the diacid form H_2_TPPS_4_^2−^, which is dominated by a B-band at 434 nm. Upon addition of increasing amounts of GQDs, the band at 434 nm decreases, without displaying any red or blue shift, and the corresponding extinction data show an almost linear behavior, with exception of the data at higher concentration of nanoparticles (Figure 11, right panel). This effect is probably due to the interference of the increasing GQDs absorption band that partially overlap with the porphyrin B-band. At the same time, a new band appears at 491 nm, accompanied by a large shoulder at longer wavelengths. This spectral feature is ascribed to the formation of J-aggregates of the parent porphyrin [63,64,65], and its concentration increases with increasing the GQD concentration (inset of Figure 11 left panel). The typical J-band in such aggregates is usually quite sharp [63] due to motional narrowing and the bandwidth is related to the coherence length [66]. In the present case, the quite broad and less intense J-band has already been reported in the case of such aggregates in the presence of polyamines or other cationic species [51,67,68]. The effect has been explained through the occurrence of a dipolar coupling mechanism very similar to what observed in colloidal metal nanoparticles and that is responsible for the enhancement of the light scattering component [69].

**Figure 12:**
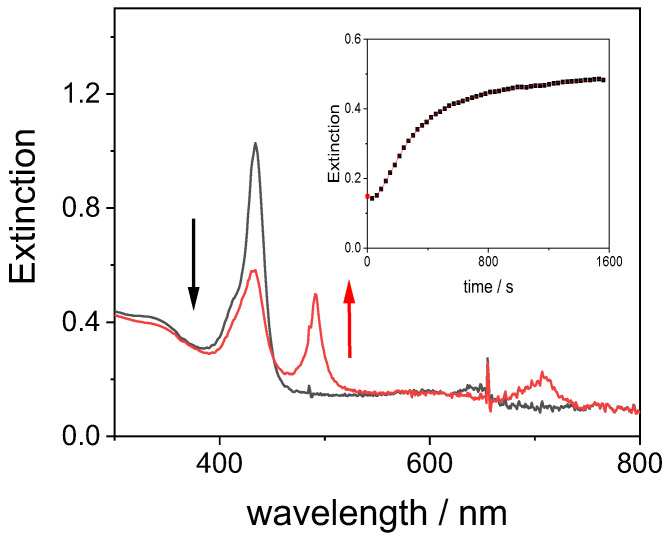
UV/Vis extinction spectra corresponding to the initial H_2_TPPS_4_^2−^ solution (black trace) and at the end of the aggregation process (red trace) after adding GQDs at pH = 3. The inset shows the kinetic trace at 491 nm for the formation of J-aggregates (from the best fitting procedure, red curve, to eq. 1, *k*_0_ = 5.1 ± 3.9 × 10^−4^ s^−1^; *k_c_* = 5.9 ± 0.2 × 10^−3^ s^−1^; m = 3.9 ± 0.6; n = 1.9 ± 0.4). Experimental conditions: [H_2_TPPS_4_^2−^] = 3 μM; [GQDs] = 0.052 mg/mL; [HCl] = 1 mM, pH = 3; T = 298 K; cell path length 1 cm.

The formation of J-aggregates has been described as a hierarchical process that strongly depends on the mixing protocol [58,67,70]. When a discrete excess of GQDs (0.052 mg/mL) is instantaneously added to the porphyrin solution, the J-aggregates form in a larger amount (Figure 12). Actually, the J-band grows gradually following a sigmoidal kinetic profile that is typical for the formation of this kind of aggregate (inset of Figure 12). The kinetic data can be analyzed through a best fitting procedure using a model developed in literature by Pasternack to describe the self-assembling process of dyes in supramolecular systems [71,72]. According to the theory, an auto-catalytic path (via *k_c_*, catalytic rate constant) depends on a characteristic power of time (*n* is a time exponent) and begins after a nucleation early stage in which a nucleus containing *m* monomers is the rate determining step. The mechanism also includes a non-catalytic pathway via *k*_0_. The values of the uncatalyzed rate constants, *k*_0_, are rather large in comparison to the catalyzed pathway (~10%). The values of *m*, the number of monomer units involved in the nucleation step, is about 4, rather close to what observed for the formation of J-aggregates when catalyzed by other cationic species [73]. We have to point out that the mixing protocol adopted in our experiments corresponds to the so-called “*porphyrin first*” or PF protocol, which should trigger aggregation under much more acidic conditions [70]. Considering that at pH = 3 the diacid porphyrin is stable with respect to aggregation, we conclude that self-assembly is triggered by the presence of GQDs. This process is reminiscent of what already reported for the J-aggregation of TPPS_4_ in the presence of gold nanorods, [74] cationic porphyrins [68], and polyamines, including *trien* [67]. Consequently, even in the present case, the capping polyamine plays an important role in favoring the self-assembling process among the monomeric porphyrin units, thus leading to the initial J-aggregates nuclei. Previous investigations have shown that the distance of the protonated amino groups in such polyamines is close to 3.8 Å, thus facilitating the stacking interactions and the electrostatic contacts among the porphine rings [67].

**Figure 13:**
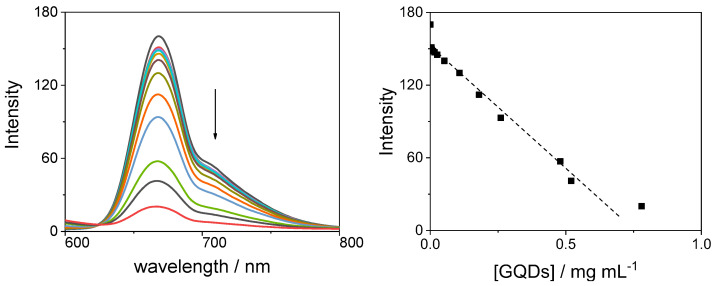
Fluorescence emission spectral changes (**left panel**) and fluorescence intensity changes at 668 nm (**right panel**) during the titration of H_2_TPPS_4_^2−^ with GQDs at pH = 3 (the arrow marks the increasing GQD concentration). Experimental conditions: [H_2_TPPS_4_^2−^] = 3 μM; [GQDs] = 0–0.78 mg/mL; [HCl] = 1 mM, pH = 3; T = 298 K; cell path length 1 cm; excitation wavelength 452 nm.

The diacid H_2_TPPS_4_^2−^ porphyrin in aqueous solution is still emissive and its emission spectra show two well-defined bands at 668 and 705 nm (Figure 13, left panel). Upon addition of increasing amounts of GQDs, the band position is not affected but the emission intensity decreases (Figure 13, right panel). This evidence is related to the formation of J-aggregates that in aqueous solution are usually not emissive or with a very short fluorescence life-times [61], due to the non-radiative pathways activated by the porphyrin-porphyrin contacts. 

To confirm the hypothesis that the observed quenching of fluorescence is simply due to the aggregation of the porphyrin, we have performed fluorescence lifetime measurements. The intensity profile for the diacid porphyrin H_2_TPPS_4_^2−^ in aqueous solution follows a mono-exponential decay with a τ_1_ = 4.0 ± 0.1 ns (A = 92%) [61]. Even in this case, a faster decaying contribution is present in our samples having a τ_2_ = 2.0 ± 0.1 ns (A = 8%), pointing to a small amount of porphyrin oligomers in solution (Table S3). Upon increasing the GQD concentration, the values of both lifetimes remain almost unaltered [62]. The measured value of the rotational correlation times for H_2_TPPS_4_^2−^ porphyrin is τ_r_ = 0.72 ns, very similar to the data reported in the literature and in agreement with the monomeric nature of this species in solution [61]. In the presence of GQDs, the values of τ_r_ increase only very slightly, indicating that the diacid porphyrins are loosely interacting with GQDs (Table S3).

Even at this pH, UV/Vis absorption and fluorescence emission titrations on the positively charged TMPyP(4)^4+^ point to the absence of any interaction (see Figure S4 and Figure S5).

## 3. Materials and Methods

### 3.1. Materials

5, 10, 15, 20-tetrakis(4-sulfonatophenyl)porphyrin (TPPS_4_), as sodium salt, and 5, 10, 15, 20-tetrakis(N-methyl-pyridinium-4-yl)porphyrin (TMPyP(4)), as tosylate salt, were received from Aldrich (Milan, Italy). All the other compounds (L-glutamic acid and triethylenetetramine tetrahydrochloride), sodium dihydrogenphosphate and sodium hydrogenphosphate, hydrochloric acid, and sodium hydroxide were of the highest commercial grade available and were used as received without further purification from Sigma-Aldrich (Milan, Italy). All the aqueous solutions were prepared in high-purity doubly distilled water (HPLC grade, Fluka, Milan, Italy). Stock solutions of the porphyrins (100–200 μM) were freshly prepared and stored in the dark to avoid photo-damage. The concentrations of the samples used in the experiments were calculated by UV/Vis absorption spectroscopy using the molar extinction coefficients at the B-band (TPPS_4_: 5.33 × 10^5^ M^−1^ cm^−1^, λ = 414 nm; TMPyP(4): 2.26 × 10^5^ M^−1^ cm^−1^, λ = 422 nm).

The GQDs were prepared by microwave assisted pyrolysis on a CEM Discover Synthesizer, Matthews, NC, U.S.A. An equimolar amount of L-glutamic acid (147 mg) and triethylenetetramine tetrahydrochloride (292 mg) was placed in a glass tube and treated for 5 min at 225 °C with an irra, diating power of 300 W. The dark residue was suspended in 1 mL of deionized water and filtered through a Millipore filter (0.45 µM) in order to remove the larger particles. The solution was then exhaustively dialyzed using tubes with a MW cut-off of 1200 Da. 

### 3.2. Methods

Fourier-transform infrared (FTIR) spectra in transmission mode were acquired in the frequency range from 4000 to 380 cm^−1^ (mid infrared region, MIR) using a Bruker Vertex 80V FTIR spectrometer. In order to obtain a good signal-to-noise ratio, each spectrum was collected at spectral resolution of 4 cm^−1^ with the co-adding and averaging of 128 scans. A background spectrum (registered in absence of sample) was collected before each measurement and subtracted from the sample spectra to eliminate the instrumental and atmospheric moisture contributions. 

Micro-Raman measurements were carried out using a LabRam HR800 Horiba spectrometer, Milan, Italy, equipped with a confocal microscope and a liquid nitrogen cooled charge coupled device (CCD) detector. The spectra were acquired in the range between 200–4000 cm^−1^ at room temperature and using a 600 gr/mm diffraction grating. The samples were excited using a laser beam with a wavelength of 532 nm that was focused by a 20× microscope objective. The laser power density, the acquisition time, and the accumulations were 5 mW, 5 s, and 20, respectively.

XPS spectra were obtained using a PHI Versa Probe II (Physical Electronics, Rome, Italy), equipped with an Al Kα (1486.6 eV) X-ray source. The survey spectra were recorded with an analyzer energy path of 117 eV, while the C1s, O1s, N1s, and Cl2p core levels were measured at 23.5 eV passing energy. The X-ray beam size was 100 microns at 25 W. A charge neutralization procedure was performed by simultaneous irradiation of samples using a low-energy electron beam and an ion beam before measuring the spectra. The position of the XPS peaks was referenced to Au metal foil (84.0 eV). XPS peaks were deconvoluted by using the Multipack Data Reduction Software (ULVAC-PHI, Inc.), employing a Shirley background curve.

UV/Vis extinction spectra were collected on an Agilent 8453 diode array spectrophotometer. Temperature was controlled at 298 K by an external water-circulating bath. 

Fluorescence emission experiments were performed on a Jasco model FP-750 spectrofluorometer equipped with a Hamamatsu R928 photomultiplier. Time-resolved fluorescence emission measurements were measured on a Jobin Yvon-Spex Fluoromax 4 spectrofluorimeter, using time-correlated single-photon counting technique. The excitation source was a NanoLED at 390 nm. Time-resolved anisotropy experiments were obtained on the same instrument using a motorized set-up of linear polarizers at the excitation and detection monochromators. 

Dynamic light scattering (DLS) measurements were performed with a Malvern Zetasizer Nano ZS equipped with a 633-nm He-Ne laser and operating at an angle of 173°. The data were collected and analysed through the Dispersion Technology Software from Malvern. For each sample, 15 runs of 10 s were acquired, with three repetitions for all the samples. The intensity size distribution, the Z-average diameter (Z-ave), and the polydispersity index (PdI) were obtained from the autocorrelation function using the “general purpose mode”.

Titration experiments were performed by collecting UV/Vis absorption and fluorescence emission spectra on solutions contained in quartz Hellma cells placed in the thermostatic holder of the instruments at 298 K. In a typical procedure, small aliquots of a stock solution containing GQDs (4 mg/mL) and porphyrin (3 µM) were progressively added to 2 mL of a prediluted TPPS_4_ 3 μM solution in a 1 cm path length cell at the pH of the experiment. The pH of the samples was controlled by phosphate buffer 1 mM or HCl (pH = 7, or 3).

The kinetic data for the aggregation of TPPS_4_ porphyrin under acidic conditions have been fitted to the equation:

Ext_t_ = Ext_∞_ + (Ext_0_ − Ext_∞_) (1 + (*m* − 1){*k*_0_t + (*n* + 1)^−1^ (*k_c_* t)*^n^*^+1^})^−1/(*m*−1)^, (1)

where Ext_0_, Ext_∞_, *k*_0_, *k_c_*, m, and n are the parameters to be optimized (Ext_t_, Ext_0_, and Ext_∞_ are the extinctions at time t, at starting time and at the end of aggregation, respectively) [71].

## 4. Conclusions

Graphene quantum dots are interesting platforms to build supramolecular systems for a variety of applications. The GQDs used in our experiments have a positively charged surface due to the presence of ionizable amino groups. At neutral pH, the tetra-anionic form of the TPPS_4_ porphyrin binds electrostatically to the nanoparticles, leading to a progressive complete quenching of its fluorescence emission. In analogy with similar systems [35,36], the deactivation mechanism could be ascribed to a photo-induced electron transfer from the electron donor excited state of the porphyrin to the acceptor GQDs. When pH is lowered and the porphyrin becomes protonated, its global charge is reduced, thus decreasing the degree of electrostatic interaction with the GQDs. At the same time, the diacid form of TPPS_4_ is able to self-aggregate and the cationic end groups of the nanoparticles catalyze the formation of J-aggregates. 

The supramolecular adduct investigated here is easy to assemble in solution and it is possible to modulate the degree of interaction among the various components changing the pH of the aqueous solution. The observed unperturbed fluorescence emission from the carbon nanomaterial could be exploited as an internal standard for ratiometric measurements. The porphyrin macrocycle offers the opportunity to exploit the coordination ability of this ligand to bind a series of metal ions, opening the way to potential application in sensing and in bio-imaging. Currently, this supramolecular assembled system is under investigation in order to develop a selective and sensitive sensor system for metal ions, by exploiting its electrochemical and fluorescence responses.

## Figures and Tables

**Table 1:** Surface composition analysis in weight % from XPS analysis on a blank sample before pyrolysis and GQDs.

Sample	O1s (wt %)	C1s (wt %)	N1s (wt %)	Cl2p (wt %)
Blank	6.37	47.74	16.46	29.43
GQDs	12.74	63.24	12.19	11.84

## Data Availability

The data are available upon request.

## Supplementary Materials

The following supporting information can be downloaded at: https://www.mdpi.com/article/10.3390/molecules29092015/s1, Figure S1. XPS spectra for Cl2p levels region on GQD; Figure S2. UV/Vis extinction spectral changes during the titration of TMPyP(4)^4+^ with GQDs at neutral pH (the arrow marks the increasing GQDs concentration). Experimental conditions: [TMPyP(4)^4+^] = 3 µM; [GQDs] = 0, 0.002, 0.01, 0.034, 0.056, 0.1, 0.18, 0.40 mg/mL; phosphate buffer 1 mM, pH = 7; T = 298 K; cell path length 1 cm; Figure S3. Fluorescence emission spectral changes during the titration of TMPyP(4)^4+^ with GQDs at neutral pH (the arrow marks the increasing GQDs concentration). The emission spectra are not corrected for the extinction of the samples. Experimental conditions: [TMPyP(4)^4+^] = 3 µM; [GQDs] = 0, 0.002, 0.01, 0.034, 0.056, 0.1, 0.18, 0.40 mg/mL; phosphate buffer 1 mM, pH = 7; T = 298 K; cell path length 1 cm; Figure S4. UV/Vis extinction spectral changes during the titration of TMPyP(4)^4+^ with GQDs at pH = 3 (the arrow marks the increasing GQDs concentration). Experimental conditions: [TMPyP(4)^4+^] = 3 µM; [GQDs] = 0, 0.01, 0.056, 0.1, –0.18 mg/mL; [HCl] = 10-3 M; T = 298 K; cell path length 1 cm; Figure S5. Fluorescence emission spectral changes during the titration of TMPyP(4)^4+^ with GQDs at pH = 3 (the arrow marks the increasing GQDs concentration). The emission spectra are not corrected for the extinction of the samples. Experimental conditions: [TMPyP(4)^4+^] = 3 µM; [GQDs] = 0, 0.01, 0.056, 0.1, −0.18 mg/mL; [HCl] = 10–3 M; T = 298 K; cell path length 1 cm; Table S1: Fluorescence lifetimes (τ_1_ and τ_2_) and relative percentage amplitudes, together with the time constant of fluorescence anisotropy decays for the titration of TPPS_4_^4−^ with GQDs at neutral pH; Table S2: Fluorescence lifetimes (τ_1_, τ_2_ and τ_3_) and relative percentage amplitudes for the titration of GQDs with TPPS_4_^4−^ at neutral pH; Table S3: Fluorescence lifetimes (τ_1_ and τ_2_) and relative percentage amplitudes, together with the time constant of fluorescence anisotropy decays for the titration of TPPS_4_^4−^ with GQDs at pH = 3.
