# Supplementary material for: Absorption and Fluorescence Emission Investigations on Supramolecular Assemblies of Tetrakis-(4-sulfonatophenyl)porphyrin and Graphene Quantum Dots
Source: Molecules. 2024 Apr 27;29(9):2015. doi: 10.3390/molecules29092015 (PMC11085775; doi:10.3390/molecules29092015)
Supplement: Supplementary file 1 [file molecules-29-02015-s001.zip › molecules-2945747-supplementary.pdf]

Supporting Information for

Absorption and Fluorescence Emission Investigations on Supramolecular Assemblies of Tetrakis-(4-sulfonatophenyl)porphyrin and Graphene Quantum Dots

Mariachiara Sarà,[1] Salvatore Vincenzo Giofrè,[1] Salvatore Abate,[1] Mariachiara Trapani,[2] Rosaria Verduci,[3] Giovanna D'Angelo,[3] Maria Angela Castriciano,[1] Andrea Romeo,[1,2] Giovanni Neri[4] and Luigi Monsù Scolaro*[1,2]

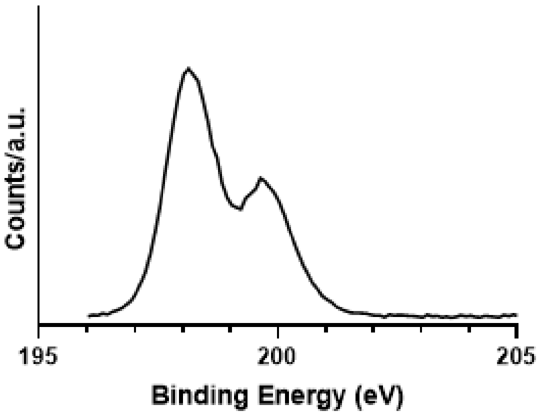

Figure S1. XPS spectra for Cl2p levels region on GQD.

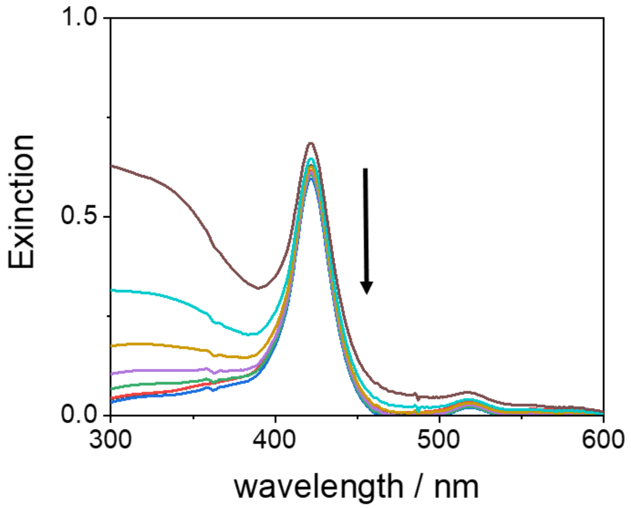

Figure S2. UV/Vis extinction spectral changes during the titration of TMPyP(4)$^{4+}$ with GQDs at neutral pH (the arrow marks the increasing GQDs concentration). Experimental conditions: [TMPyP(4)$^{4+}$] = 3 μM; [GQDs] = 0, 0.002, 0.01, 0.034, 0.056, 0.1, 0.18, 0.40 mg/mL; phosphate buffer 1 mM, pH = 7; T = 298 K; cell path length 1 cm.

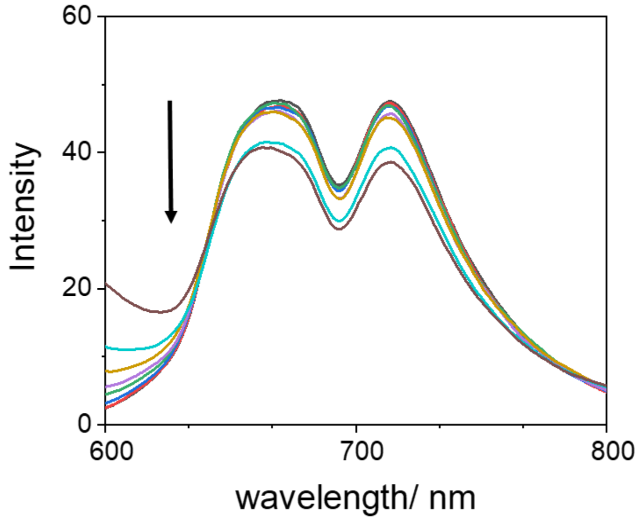

Figure S3. Fluorescence emission spectral changes during the titration of TMPyP(4)$^{4+}$ with GQDs at neutral pH (the arrow marks the increasing GQDs concentration). The emission spectra are not corrected for the extinction of the samples. Experimental conditions: [TMPyP(4)$^{4+}$] = 3 μM; [GQDs] = 0, 0.002, 0.01, 0.034, 0.056, 0.1, 0.18, 0.40 mg/mL; phosphate buffer 1 mM, pH = 7; T = 298 K; cell path length 1 cm.

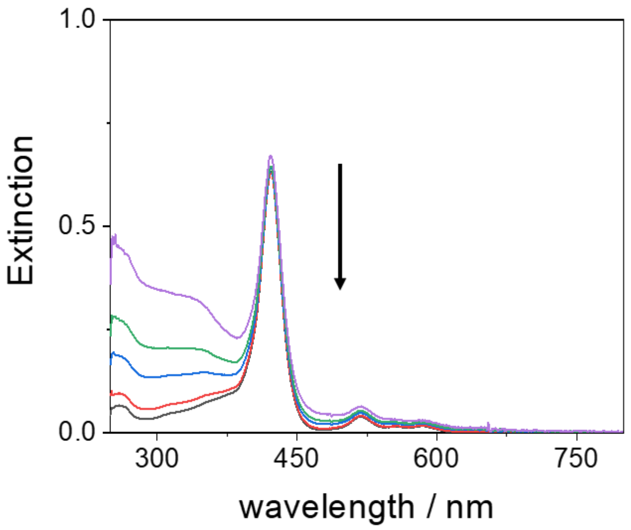

Figure S4. UV/Vis extinction spectral changes during the titration of TMPyP(4)$^{4+}$ with GQDs at pH = 3 (the arrow marks the increasing GQDs concentration). Experimental conditions: [TMPyP(4)$^{4+}$] = 3 μM; [GQDs] = 0, 0.01, 0.056, 0.1, – 0.18 mg/mL; [HCl] = 10^{-3} M; T = 298 K; cell path length 1 cm.

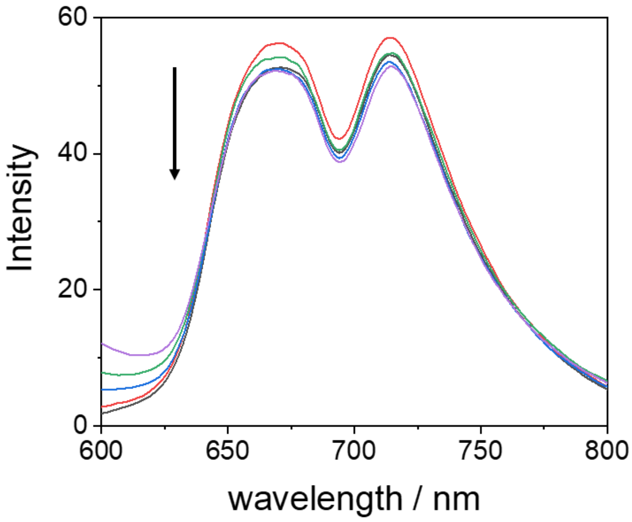

Figure S5. Fluorescence emission spectral changes during the titration of TMPyP(4)$^{4+}$ with GQDs at pH = 3 (the arrow marks the increasing GQDs concentration). The emission spectra are not corrected for the extinction of the samples. Experimental conditions: [TMPyP(4)$^{4+}$] = 3 μM; [GQDs] = 0, 0.01, 0.056, 0.1, – 0.18 mg/mL; [HCl] = 10^{-3} M; T = 298 K; cell path length 1 cm.

Table S1. Fluorescence lifetimes (τ_1 and τ_2) and relative percentage amplitudes, together with the time constant of fluorescence anisotropy decays for the titration of $TPPS_4^{4-}$ with GQDs at neutral pH. Experimental conditions: [$TPPS_4^{4-}$] = 3 μM; phosphate buffer 10 mM, pH = 7; T = 298 K; cell path length 1 cm; λ_{exc} = 390 nm; λ_{em} = 644 nm.

**[GQDs] / mg mL^{-1}**	**Lifetime / ns (relative amplitude)**		**Anisotropy Decays / ns**
0	τ_1 = 10.0 ± 0.1 (97%)	τ_2 = 1.1 ± 0.1 (3%)	τ_r = 0.72 ± 0.1
0.02	τ_1 = 10.4 ± 0.1 (93%)	τ_2 = 1.3 ± 0.1 (7%)	τ_r = 0.96 ± 0.1
0.04	τ_1 = 9.99 ± 0.1 (92%)	τ_2 = 1.4 ± 0.1 (8%)	τ_r = 1.00 ± 0.1
0.06	τ_1 = 10.1 ± 0.1 (86%)	τ_2 = 1.8 ± 0.1 (14%)	τ_r = 0.92 ± 0.1

Table S2. Fluorescence lifetimes ($\tau_1\tau$,$_2$ and τ_3) and relative percentage amplitudes for the titration of GQDs with $TPPS_4^{4-}$ at neutral pH. Experimental conditions: [GQDs] = 0.14 mg/mL; phosphate buffer 10 mM, pH = 7; T = 298 K; cell path length 1 cm; λ_{exc} = 390 nm; λ_{em} = 474 nm.

**[$TPPS_4$] / μM**	**Lifetime / ns (relative amplitude)**		
0	τ_1= 15.4 ± 0.1 (14%)	τ_2=5.4 ± 0.1 (61%)	τ_3=1.8 ± 0.1 (25%)
0.07	τ_1= 14.6 ± 0.1 (17%)	τ_2=5.0 ± 0.1 (64%)	τ_3=1.5 ± 0.1 (19%)
0.42	τ_1= 14.9 ± 0.1 (16%)	τ_2=5.2 ± 0.1 (72%)	τ_3=1.6 ± 0.1 (22%)
0.90	τ_1= 15.5 ± 0.1 (15%)	τ_2=5.3 ± 0.1 (61%)	τ_3=1.8 ± 0.1 (24%)
1.80	τ_1= 16.3 ± 0.1 (14%)	τ_2=5.4 ± 0.1 (64%)	τ_3=1.7 ± 0.1 (22%)
3.00	τ_1= 16.5 ± 0.1 (15%)	τ_2=5.6 ± 0.1 (63%)	τ_3=1.9 ± 0.1 (22%)

Table S3. Fluorescence lifetimes (τ_1 and τ_2) and relative percentage amplitudes, together with the time constant of fluorescence anisotropy decays for the titration of $TPPS_4^{4-}$ with GQDs at pH = 3. Experimental conditions: [$TPPS_4^{4-}$] = 3 μM; pH = 3 ([HCl] = 10^{-3} M); T = 298 K; cell path length 1 cm; λ_{exc} = 390 nm; λ_{em} = 670 nm.

**[GQDs] / mg mL^{-1}**	**Lifetime / ns (relative amplitude)**		**Anisotropy Decays / ns**
0	τ_1 = 3.9 ± 0.1 (92%)	τ_2 = 1.9 ± 0.1 (8%)	τ_r = 0.88 ± 0.1
0.02	τ_1 = 3.8 ± 0.1 (90%)	τ_2 = 1.9 ± 0.1 (10%)	τ_r = 0.99 ± 0.1
0.08	τ_1 = 3.8 ± 0.1 (90%)	τ_2 = 1.9 ± 0.1 (10%)	τ_r = 1.02 ± 0.1
0.28	τ_1 = 3.9 ± 0.1 (92%)	τ_2 = 1.9 ± 0.1 (10%)	τ_r = 1.05 ± 0.1
